# Drivers of the Sex Disparity in Statin Therapy in Patients with Coronary Artery Disease: A Cohort Study

**DOI:** 10.1371/journal.pone.0155228

**Published:** 2016-05-05

**Authors:** Huabing Zhang, Jorge Plutzky, Maria Shubina, Alexander Turchin

**Affiliations:** 1 Department of Endocrinology, Key Laboratory of Endocrinology, Ministry of Health, Peking Union Medical College Hospital, Chinese Academy of Medical Sciences, Beijing, China; 2 Harvard Medical School, Boston, Massachusetts, United States of America; 3 Brigham and Women's Hospital, Boston, Massachusetts, United States of America; 4 Harvard Clinical Research Institute, Boston, Massachusetts, United States of America; Universite de Montreal, CANADA

## Abstract

**Background:**

Women are less likely to be prescribed statins than men. Existing reports explain only a fraction of this difference. We conducted a study to identify factors that account for sex differences in statin therapy among patients with coronary artery disease (CAD).

**Methods and Results:**

We retrospectively studied 24,338 patients with CAD who were followed for at least a year between 2000 and 2011 at two academic medical centers. Women (9,006 / 37% of study patients) were less likely to either have initiated statin therapy (81.9% women vs. 87.7% men) or to have persistent statin therapy at the end of follow-up (67.0% women vs. 71.4% men). Women were older (72.9 vs. 68.4 years), less likely to have ever smoked (49.8% vs. 65.6%), less likely to have been evaluated by a cardiologist (57.5% vs. 64.5%) and more likely to have reported an adverse reaction to a statin (27.1% vs. 21.7%) (p < 0.0001 for all). In multivariable analysis, patients with history of smoking (OR 1.094; p 0.017), younger age (OR 1.013 / year), cardiologist evaluation (OR 1.337) and no reported adverse reactions to statins (OR 1.410) were more likely (p < 0.0001 for all) to have persistent statin therapy. Together, these four factors accounted for 90.4% of the sex disparity in persistent statin therapy.

**Conclusions:**

Several specific factors appear to underlie divergent statin therapy in women vs. men. Identifying such drivers may facilitate programmatic interventions and stimulate further research to overcome sex differences in applying proven interventions for cardiovascular risk reduction.

## Introduction

Coronary artery disease (CAD) is the leading cause of death and disability in both men and women in United States and many developed countries.[1–3] Although on average the onset of CAD is about 10 years later in women than in men, women carry a greater burden of cardiovascular risk factors and have higher prevalence of symptoms, myocardial ischemia, and mortality relative to men.[4,5] While mortality rates from CAD have fallen significantly in both men and women over the past 30 years, this decline has been much more dramatic among men.[2] The reason for this difference is multifactorial, but lower adherence to evidence-based guidelines in women may be a key contributor.[6,7]

Statins are highly effective in decreasing the risk of secondary coronary events[8–10] and they are equally effective in men and women.[11–13] Consequently consensus guidelines recommend statin therapy for all adult patients with CAD irrespective of sex.[1,8] Nevertheless women are less likely to be prescribed statins than men in patients with CAD.[14–28] The reasons for the sex disparity in statin therapy are not clear. Prior reports suggest that patients' characteristics including demographics, economic status, physical health status, depression and lifestyle risk factors account for less than one third of the difference in statin use. Among women and men, Latino race/ethnicity, lack of prescription drug coverage and greater functional disability were negatively associated with statin use. African American race and low educational status were negatively associated with statin use in men alone. There was a higher likelihood of statin use among both women and men who had fair health status, those who were overweight or obese, and among men who were smokers in the past (but not current smokers). [26] We therefore conducted a retrospective cohort study to evaluate the contribution of age, smoking history, evaluation by a cardiologist and history of reported adverse reactions to statins to sex disparities in statin therapy in patients with CAD.

## Methods

### Design

In order to identify factors that could explain the difference in statin therapy between women and men, we first established patient and treatment characteristics that were associated with statin therapy in patients with CAD. Among the characteristics that were significantly associated with higher probability of statin therapy, we then identified the ones that had different distribution between men and women. As the final step, we calculated the relative contributions of the characteristics that had different prevalence in women vs. men to the sex disparities in statin therapy.

### Study Cohort

Study participants included adult patients (> = 18 years old) with CAD followed in primary care practices affiliated with Brigham and Women’s Hospital (BWH) or Massachusetts General Hospital (MGH) for at least a year during the study period from January 1st, 2000 to December 31st, 2011. Treatment in a primary care practice was defined as having notes in a primary care clinic on at least two distinct dates during the study period. Patients were excluded from the study if demographic information (sex and median income by zip code) was not available.

For an individual patient study entry date was defined as the later of a) date of diagnosis of CAD and b) the first primary care practice note after January 1st, 2000. We included only patients who had notes in a primary care clinic for at least 1 year after the study entry date in order to ensure sufficient data for analysis. Date of the last note in a primary care clinic during the study period served as the study exit date. This study was approved by Partners Human Research Committee which is the institutional review board at the Partners HealthCare System and the requirement for written informed consent was waived. Patient records were de-identified and analyzed anonymously.

### Study Measurements

An individual patient served as the unit of analysis. Statin use was defined as at least one statin prescription during study period. Patient was categorized as having persistent statin therapy if they had an active statin electronic medical record (EMR) medication record upon study exit (defined as a statin medication record updated within twelve months prior to the study exit without a subsequent explicit discontinuation).

Patient age was calculated at study entry. Diagnoses of CAD and diabetes mellitus (DM), history of coronary artery bypass grafting (CABG), percutaneous coronary intervention (PCI) and myocardial infarction (MI), and family history of CAD were established from the EMR data. The definition of CAD was based on the diagnosis of CAD or MI on the problem list or history of CABG or PCI. Highest low-density lipoprotein cholesterol (LDL-C) level was defined as the highest LDL-C recorded before the study exit. Baseline body mass index (BMI) and smoking status were identified from the EMR records prior to the study entry. Patient was categorized as having been evaluated by a cardiologist if they had at least one note in a cardiology clinic within the Partners system during the study period. Information on reported adverse reactions to statins (clinical events / symptoms documented by healthcare providers as thought to have been caused by a statin) was obtained from a combination of structured EMR data and computational processing of narrative electronic provider notes using specially designed natural language processing software (Canary). The software utilizes a language model of documentation of clinical events related to medications that includes over 1,200 rules. These rules recognize clinical events that are etiologically linked in the text to a set of specific medications (e.g. "Lipitor") or classes (e.g. "statins"). While the software can recognize documentation of clinical events related to any set of medications, it was specifically validated for identification of adverse reactions to statins, whereupon it achieved sensitivity of at least 86.5% and specificity of at least 91.9%[29]. The software is available from the authors upon request.

Demographic information, medication and laboratory data were obtained from the EMR at Partners HealthCare System—an integrated health care delivery network in eastern Massachusetts that includes BWH and MGH. The Partners HealthCare EMR system was fully integrated by 2000, so includes all prescription and laboratory records for patients over the study period. No changes were made to these systems over the 12 years of this study.

### Statistical Analysis

Summary statistics were calculated using frequencies and proportions for categorical data and means (SDs), medians, and ranges for continuous variables. Quantitative variables were compared across multiple patient categories using one-way ANOVA and categorical variables using chi-square. A multivariable logistic regression model was used to identify patient characteristics associated with use or persistence of statin therapy, compared to no statin therapy or non-persistent use of statins. Patient demographics (age, sex, race, health insurance, primary language, and median income by zip code), diagnosis of diabetes mellitus, family history of CAD, history of smoking, maximum LDL level, baseline BMI, cardiologist evaluation and reported adverse reactions (only in the analysis of persistence of statin therapy) were included as covariates in the analysis. Primary language was represented as a binary variable (“English” vs. “Other”). Multiple imputation technique was used to account for missing data for all variables that had missing information for at least one patient (smoking status, maximum LDL level and baseline BMI). For variables that describe diagnoses (e.g. CABG, MI) absence of information was interpreted as absence of diagnosis, since absence of diagnosis is not routinely recorded in the EMR. The analysis was adjusted for clustering within primary care providers. Clustering adjustment was done using GEE (generalized estimating equations) models. Thresholds for statistical significance were adjusted for multiple hypothesis testing using Simes-Hochberg method[30,31].

Candidate variables were selected a priori based on their perceived clinical relevance. Additionally, all available demographic data were included.

To estimate the impact of a covariate or a group of covariates of interest on sex differences in probabilities of a binary event of interest (statin use or persistence of statin therapy for statin users), we directly standardized (re-weighted) the distribution of a covariate or a group of covariates of women to those of men to estimate adjusted probabilities of the event for women[32]. Direct standardization was also applied for estimation of adjusted probability of persistence of statin therapy in all study population for covariates which were defined for all subjects. To estimate adjusted probabilities for covariates which are pertinent only to statin users, we used two level direct standardization: first by covariates defined for every subject, then for remaining covariates within each stratum defined by the first group of covariates.

The adjusted sex disparity was defined as the difference between the observed probability of the event for men and the adjusted probability of the event for women. Disparity explained by a covariate or a set of covariates was defined as the difference between the observed disparity and adjusted disparity, and the disparity fraction explained by the covariates of interest was calculated as a ratio of the explained disparity to observed disparity.

We applied direct standardization to estimate adjusted probabilities and fractions of sex disparity in statin use explained by cardiology evaluation, smoking and age group for each covariate and jointly by all three. For statin users direct standardization was applied to estimate adjusted probabilities and fractions of sex disparity in persistence in statin therapy explained univariately by cardiology evaluation, history of smoking, and age group. Two-level direct standardization was used to calculate the univariate contribution of adverse reaction attributed to statin as well as the joint contribution of all four variables. Bootstrap method, stratified by sex, was used to estimate variances for adjusted probabilities, adjusted and explained disparities and disparity fractions explained by covariates[33]. All data was analyzed using SAS, version 9.3 (SAS Institute, Cary, North Carolina). The authors had full access to and take full responsibility for the integrity of the data. All authors have read and agree to the manuscript as written.

## Results

### Study Cohort

We identified 24,809 patients with a diagnosis of CAD who were followed for at least 1 year by BWH or MGH primary care physicians between 2000 and 2010. We excluded 471 patients who had missing demographics information; the remaining 24,338 patients were included in the analysis (Table 1). Two thirds of the patients were men; 20,830 (85.6%) received statins during the study period (Table 2) and 16,792 (69.7%) had persistent statin therapy.

**Table 1:** Characteristics of all study patients by sex. Abbreviations: LDL-C, low density lipoprotein-cholesterol; BMI, body mass index; CAD, coronary artery disease.

Variable	Number of patients with missing information (%)	Male	Female	P-value
**Study patients, n (%)**		**15,332 (63.0%)**	**9,006 (37.0%)**	
**Age, mean (± SD), years**	**0 (0)**	**68.4 (11.8)**	**72.9 (12.1)**	**< 0.001**
**Race/Ethnicity n (%)**				
** White**		**13,124 (85.6%)**	**7,312 (80.7%)**	**< 0.001**
** Black**		**385 (2.5%)**	**467 (5.2%)**	
** Hispanic**		**381 (2.5%)**	**327 (3.6%)**	
** Asian**		**214 (1.4%)**	**93 (1.0%)**	
Other*		**1,228 (8.0%)**	**807 (9.0%)**	
**Median income by zip code, mean (± SD), $1,000s**	**0 (0)**	**73.4 (27.7)**	**68.9 (27.4)**	**< 0.001**
**English as primary language**	**0 (0)**	**13,776 (89.9%)**	**7,817 (86.8%)**	**< 0.001**
**Government insurance, n (%)**	**0 (0)**	**10,494 (69.5%)**	**7,137 (79.3%)**	**< 0.001**
**History of smoking, n (%)**	**2,034 (8.4)**	**10,053 (65.6%)**	**4,487 (49.8%)**	**< 0.001**
**Diabetes mellitus, n (%)**	**0 (0)**	**6,424 (41.9%)**	**3,661 (40.7%)**	**0.056**
**Family history of CAD, n (%)**	**0 (0)**	**2,286 (14.9%)**	**1,361 (15.1%)**	**0.670**
**Maximum LDL-C, mg/dl, mean (SD)**	**2,202 (9.0)**	**127.4 (42.1)**	**137.2 (46.6)**	**< 0.001**
**Baseline BMI, kg/m**^**2**^**, mean (SD)**	**3,513 (14.4)**	**29.6 (5.3)**	**29.7 (6.7)**	**0.595**
**Cardiologist evaluation, n (%)**	**0 (0)**	**9,889 (64.5%)**	**5,179 (57.5%)**	**< 0.001**
**Coronary artery bypass grafting, n (%)**	**0 (0)**	**2,602 (17.0%)**	**911 (10.1%)**	**< 0.001**
**Myocardial infarction, n (%)**	**0 (0)**	**5,789 (37.8%)**	**3,311 (36.8%)**	**0.122**
**Use of statin therapy, n (%)**	**0 (0)**	**13,453 (87.7%)**	**7,377 (81.9%)**	**< 0.001**
**Persistent statin therapy, n (%)**	**0 (0)**	**10,940 (71.4%)**	**6,032 (67.0%)**	**< 0.001**

*Includes unknown

**Table 2:** Characteristics of all study patients treated with a statin, by sex. Abbreviations: LDL-C, low density lipoprotein-cholesterol; BMI, body mass index; CAD, coronary artery disease.

Variable	Number of patients with missing information (%)	Male	Female	P-value
**Study patients, n (%)**		**13,453 (64.6%)**	**7,377 (35.4%)**	
**Age, mean (± SD), years**	**0 (0)**	**67.9 (11.5)**	**72.3 (11.5)**	**< 0.001**
**Race/Ethnicity n (%)**				
** White**		**11,612 (86.3%)**	**6,032 (81.8%)**	**< 0.001**
** Black**		**330 (2.5%)**	**397 (5.4%)**	
** Hispanic**		**340 (2.5%)**	**290 (3.9%)**	
** Asian**		**187 (1.4%)**	**77 (1.0%)**	
Other*		**984 (7.3%)**	**581 (7.9%)**	
**Median income by zip code, mean (± SD), $1,000s**	**0 (0)**	**73.4 (27.6)**	**68.9 (27.4)**	**< 0.001**
**English as primary language**	**0 (0)**	**12,144 (90.3%)**	**6,432 (87.2%)**	**< 0.001**
**Government insurance, n (%)**	**0 (0)**	**9,107 (67.8%)**	**5,837 (79.1%)**	**< 0.001**
**History of smoking, n (%)**	**1,389 (6.7)**	**9,009 (67.0%)**	**3,860 (52.3%)**	**< 0.001**
**Diabetes mellitus, n (%)**	**0 (0)**	**5,717 (42.5%)**	**3,205 (43.5%)**	**0.185**
**Family history of CAD, n (%)**	**0 (0)**	**2,100 (15.6%)**	**1,186 (16.1%)**	**0.376**
**Maximum LDL-C, mg/dl, mean (SD)**	**1,578 (7.6)**	**128.0 (42.2)**	**138.1 (47.3)**	**< 0.001**
**Baseline BMI, kg/m**^**2**^**, mean (SD)**	**2,541 (12.2)**	**29.7 (5.3)**	**30.0 (6.7)**	**0.005**
**Cardiologist evaluation, n (%)**	**0 (0)**	**9,073 (67.4%)**	**4,570 (62.0%)**	**< 0.001**
**Coronary artery bypass grafting, n (%)**	**0 (0)**	**2,374 (17.7%)**	**817 (11.1%)**	**< 0.001**
**Myocardial infarction, n (%)**	**0 (0)**	**5,200 (38.7%)**	**2,825 (38.3%)**	**0.611**
**Reported adverse reactions, n (%)**	**0 (0)**	**2,913 (21.7%)**	**2,000 (27.1%)**	**< 0.001**
**Persistent statin therapy, n (%)**	**0 (0)**	**10,940 (81.3%)**	**6,032 (81.8%)**	**0.427**

*Includes unknown

In univariate analysis, women were less likely than men to have been treated with a statin but equally likely to have persistent statin therapy over the course of the study. Women were older, less likely to have ever smoked, less likely to have been evaluated by a cardiologist and more likely to have reported an adverse reaction to a statin. (Tables 1 & 2)

### Predictors for statin use and persistence

In multivariable analysis, diagnosis of diabetes, family history of CAD, younger age, history of smoking and cardiologist evaluation were associated with greater likelihood of statin use (Table 3) while female sex was associated with lower likelihood. In multivariable analysis in patients who had initiated statin therapy, diagnosis of diabetes, family history of CAD, female sex, younger age, history of smoking, and cardiologist evaluation were associated with greater likelihood of persistent statin therapy, while reported adverse reactions to statins were associated with lower likelihood of remaining on statin treatment (Table 4). There were no significant differences in distribution of diabetes or family history of CAD between men and women (Tables 1 & 2). On the other hand, women were significantly older, less likely to smoke, less likely to have been evaluated by a cardiologist and more likely to have reported an adverse reaction to a statin than men (Tables 1 & 2). These four patient and treatment characteristics were therefore subsequently examined as possible candidates for the explanation of the differences in statin therapy between women and men. We have also conducted a sensitivity analysis excluding patients with race equal to "Other", with the results similar to the original analysis.

**Table 3:** Effects of patient characteristics on statins use (a multivariable analysis). Abbreviations: CAD, coronary artery disease; LDL-C, low density lipoprotein-cholesterol; BMI, body mass index.

Variable	Odds Ratio	95% Confidence Limits		P-value
**Age, per 1 year increase**	**0.979**	**0.975**	**0.984**	**< .001**
**Female**	**0.771**	**0.715**	**0.831**	**< .001**
**Race / Ethnicity (compared to Caucasian)**				
** African-American**	**0.863**	**0.708**	**1.052**	**0.145**
** Hispanic**	**1.094**	**0.836**	**1.432**	**0.513**
** Asian**	**0.928**	**0.689**	**1.251**	**0.624**
Other*	**0.718**	**0.629**	**0.820**	**< .001**
**Income, per $1,000 increase**	**1.002**	**1.000**	**1.003**	**0.020**
**Government insurance**†	**1.098**	**1.003**	**1.203**	**0.043**
**English as the first language**	**0.996**	**0.873**	**1.135**	**0.950**
**Diabetes mellitus**	**1.376**	**1.272**	**1.489**	**< .001**
**Family history of CAD**	**1.371**	**1.212**	**1.551**	**< .001**
**Ever smoker**	**1.144**	**1.061**	**1.234**	**0.001**
**Baseline BMI, per 1kg/m**^**2**^ **increase**	**1.014**	**1.005**	**1.022**	**0.001**
**Maximum LDL level, per 10mg/dl increase**	**1.015**	**1.004**	**1.025**	**0.007**
**Evaluation by cardiologist**	**2.535**	**2.339**	**2.747**	**< .001**

*Includes unknown

†Compared to non-government insurance

**Table 4:** Effects of patient characteristics on persistence of statin therapy (a multivariable analysis). Abbreviations: CAD, coronary artery disease; LDL-C, low density lipoprotein-cholesterol; BMI, body mass index.

Variable	Odds Ratio	95% Confidence Limits		P-value
**Age, per 1 year increase**	**0.987**	**0.983**	**0.991**	**< .0001**
**Female**	**1.134**	**1.051**	**1.224**	**0.001**
**Race / Ethnicity (compared to Caucasian)**				
** African-American**	**1.014**	**0.838**	**1.228**	**0.886**
** Hispanic**	**1.219**	**0.948**	**1.567**	**0.123**
** Asian**	**1.213**	**0.864**	**1.701**	**0.265**
Other*	**0.780**	**0.684**	**0.889**	**0.000**
**Income, per $1,000 increase**	**0.998**	**0.997**	**1.000**	**0.017**
**Government insurance**†	**1.025**	**0.943**	**1.114**	**0.560**
**English as the first language**	**0.988**	**0.878**	**1.111**	**0.838**
**Diagnosis of diabetes**	**1.164**	**1.084**	**1.249**	**< .0001**
**Family history of CAD**	**1.363**	**1.233**	**1.507**	**< .0001**
**Ever smoker**	**1.094**	**1.016**	**1.177**	**0.017**
**Baseline BMI, per 1kg/m**^**2**^ **increase**	**1.004**	**0.997**	**1.011**	**0.245**
**Maximum LDL level, per 10mg/dl increase**	**0.989**	**0.980**	**0.997**	**0.009**
**Evaluation by cardiologist**	**1.337**	**1.237**	**1.446**	**< .0001**
**Reported adverse reactions**	**0.709**	**0.654**	**0.768**	**< .0001**

*Includes unknown

†Compared to non-government insurance

### Contribution of age, smoking, cardiologist evaluation and reported adverse statin reactions to sex differences in statin use

Direct estimates of the effect of differences in joint distributions of covariates between two groups can be used to assess the relative contributions of specific covariates to these differences. In applying this method to differences in statin use between men and women, the following contributions were identified: age 29.5%, smoking 22.1%, and cardiologist evaluation 17.9%. Together, these three factors contributed 48.9% to the sex disparity in statin use. Patients with possible evidence of more severe CAD were more likely to be seen by a cardiologist. Among patients with history of CABG, 78.5% were evaluated by a cardiologist while only 59.1% of patients without history of CABG received evaluation by a cardiovascular specialist. Cardiologists saw 68.6% of patients with history of MI while only 57.9% of patients without history of MI were evaluated by a cardiologist. However, this difference in CAD severity accounted only for a fraction of the contribution of cardiologist evaluation to the sex disparity. Including history of CABG and MI in the model only reduced the contribution of cardiologist evaluation to 13.2%.

Direct estimates of the effect of the differences in joint distributions of the covariates between men and women showed the following contributions to sex differences in persistent statin therapy: age 51.5%, smoking 31.9%, cardiologist evaluation 25.1%, and reported adverse statin reactions 6.3%. Together, these four factors accounted for 90.4% of the sex disparity in persistent statin therapy.

## Discussion

In this large retrospective study we found that only 85.6% of the patients with a diagnosis of CAD ever received statins during the study period and less than 70% had persistent therapy by the end of follow-up. Importantly, four patient characteristics–age, smoking history, evaluation by a cardiologist and reported adverse reactions to statins–underlie most of the sex differences in persistent statin therapy. This is the first study that was able to identify patient characteristics and other factors that account for a large fraction of the sex disparity in statin therapy. These findings therefore have significant public health implications by pointing out both areas where interventions might be applied to reduce sex disparities in statin therapy as well as important population differences between women and men with CAD (e.g. in age and rates of smoking) that may be giving rise to a large component of the apparent disparity.

The present study has several strengths. Access to EMR data from two large hospital systems allowed us to analyze data on more than 24,000 patients with CAD with diverse backgrounds. The EMR data enabled us to analyze a comprehensive dataset that included laboratory data, body mass index, family history and smoking status, which are generally not available in claims data. Use of a specially designed natural language processing software identified a large number of reported adverse reactions to statins that were only documented in provider notes. Finally, we were able to follow the patients longitudinally providing a more comprehensive assessment compared to a cross-sectional analysis.

Current guidelines recommend statin therapy for all adult patients with CAD irrespective of age based on randomized placebo-controlled evidence for statin benefits across various clinical profiles.[8,9] The guidelines for treatment of patients with CAD didn’t change significantly over the study period.[34,35] In clinical trials statin therapy reduces the risk of cardiovascular events in patients with CAD older than 75 years of age. [36,37] However, acknowledging that older participants in clinical trials were likely to be healthier than many older individuals in the general population, guidelines recommend that the use of statin therapy should be individualized in persons with CAD >75 years of age. [8] In clinical practice, older people are thought to be less likely to be on statins. [38,39] Our study supports this finding of decreased persistence of statin therapy as a function of age. Women in our study were on average 4.5 years older than men–in keeping with the known later onset of CAD in women– 48% of them were older than 75. Thus the older age of CAD onset in women may have contributed to the sex disparities of statin therapy in our study. The fact that statin therapy in older individuals remains a matter of debate could be an explanation for this finding.

Smoking is a well-known risk factor for cardiovascular events.[40,41] However, all patients in our analysis already had a documented diagnosis of CAD and therefore were at sufficiently high risk of cardiovascular events to justify statin therapy[8]. Nevertheless, other investigators have also observed that in clinical practice patients at higher cardiovascular risk are more likely to receive statin therapy.[24,42] This approach may be supported by clinical trial evidence that smokers as a subgroup are amongst those benefiting the most from statin use. [12] Since smoking rates are higher in men than women [2], tobacco use may drive greater statin use in men versus women as identified in this study and also seen by others. [26]

We found that women were less likely to be evaluated by a cardiologist, which may contribute to sex disparity in statin use given that cardiologist evaluation contributes to statin use in general[43]. Prior studies support the notion that women are less likely to see a cardiovascular specialist although this has not been directly linked to differences in statin use.[44] While it is possible that cardiologists are more aggressive in prescribing statins because their patients have a higher risk profile, our findings were not consistent with this explanation. Even though patients evaluated by a cardiologist were more likely to have a history of MI or CABG, these factors accounted for only a fraction (4.7% out of 17.9%) of the sex disparity in statin therapy explained by differences in cardiology evaluation. Specialist consultation may narrow the gap in clinical performance measures between women and men [44–46] suggesting a role of referrals in improving outcomes and mitigating performance disparities. Other studies have suggested that management of hypertension and other cardiovascular parameters may be more aggressive and effective when done by a cardiologist rather than an internist.[44,45] Given the potential impact on appropriate use, further research is needed to establish the reasons for both this unexplained sex disparity in cardiology referrals as well as the under-prescribing patterns among primary care physicians. At the same time, we must bear in mind that "evaluation by a cardiologist" includes not just the actions by cardiologists but also referral patterns by PCPs and patients' acceptance of such referrals, all of which could be contributing to the sex differences in statin therapy.

Adverse reactions are commonly reported by patients receiving statins in clinical setting as opposed to clinical trials.[47,48] In our study women were more likely than men to report adverse reactions to statins, as also reported by others [49–51]. The difference in reported adverse events to statin treatment was a significant contributor to the sex disparity in statin therapy. The reasons for differences in frequency of reported adverse reactions to statins between men and women require further exploration. Several known risk factors for adverse reactions to statins may contribute to these differences, including older age, more metabolic syndrome factors, smaller body size, etc.[52]

Successful statin therapy in appropriate patients involves two components: initiation and persistence. We found that women were less likely to have been ever used statin therapy than men. However, women already on statin therapy were more likely to persist on it. The reasons for this difference are uncertain, and existing literature on statin therapy adherence in women is mixed.[53,54] More research is needed to gain better understanding of sex differences in persistence on statin therapy.

When effects of the differences in initiation and persistence were combined, women were less likely to receive statin treatment overall. In our study, women were 6.6% less likely to have ever used statins and 6.2% less likely to have persistent statin therapy, comparable with previously published research. [14–27] Less frequent preventive therapy recommendations by physicians for women have been attributed to the lower perceived CVD risk for women by the physicians, despite the evidence demonstrating statin efficacy in decreasing the risk of secondary CVD events in both women and men.[55] These findings suggest the clinicians’ assumptions regarding cardiovascular risk in women require further consideration and attention.

It is important to note that a degree of controversy continues to exist about the benefits women derive from statin therapy. A recent meta-analysis focused on secondary prevention and limited to placebo-controlled trials confirmed a reduction in cardiovascular events similar to men but did not find a benefit in all-cause mortality or stroke.[56] On the other hand, a larger meta-analysis that included primary prevention and “usual care” controlled trials found a similar reduction in all-cause mortality in both women and men. The debate continues about the relative merits of these analyses[57] and consequently some clinicians may remain unconvinced about the benefits of statin therapy in women, leading to lower treatment rates. However, in the secondary prevention population that was the focus of our study, the reduction in the incidence of cardiovascular events–an important clinical endpoint—has been a consistent finding.[13,56] Consequently, the most recent AHA / ACC as well as ESC / EAS guidelines recommend statin therapy for secondary prevention in both women and men ≤ 75 years of age, with the evidence rating of NHLBI Grade A or AHA/ACC Class of Recommendation I (highest in both cases). [58,59]

Our findings must be interpreted in the light of several limitations. Our analysis was observational in nature and therefore could only establish associations rather than causal relationships. Our study population included patients from two primarily academic hospitals in eastern Massachusetts, so the results may not be generalizable to patients in other settings. Information on patients who received some of their care outside of Partners HealthCare may have been incomplete. We did not have the information of the maximum LDL while off statin and the number of years since CAD diagnosis and could not assess their contribution to statin therapy. Not all patients in the study may have had clinical ASCVD–for example, some patients who had a diagnosis of “CAD” recorded in the electronic medical records could have had asymptomatic partial occlusion of coronary arteries identified on angiography. Accuracy of the natural language processing algorithm while high, was not perfect and some adverse reactions may have been missed. Finally, EMR medication data may not comprehensively reflect statin utilization by patients.

In summary, in 24,809 patients with CAD in whom indications for statin therapy were unequivocal, a discrete set of four specific factors appear to account for the majority of differences between men and women in statin use and persistent use. Our data reveals significant issues and opportunities for improving cardiovascular outcomes in women by eliminating sex disparity in statin use in scenarios where their clinical benefits are indisputable. As an example, statin rechallenge after an adverse reaction could be a potential strategy for the practicing clinician to decrease sex disparity in statin therapy. Identifying drivers for decreased appropriate statin use in women may facilitate interventions and stimulate research to overcome sex differences in applying proven interventions for cardiovascular risk reduction.
